# Novel versions of Hölder's-Like and related inequalities with newly defined $L_{P}$ space, and their applications over fuzzy domain

**DOI:** 10.1016/j.heliyon.2024.e40664

**Published:** 2024-11-27

**Authors:** Xiangting Shi, Ahmad Aziz Al Ahmadi, Muhammad Bilal Khan, Loredana Ciurdariu, Khalil Hadi Hakami

**Affiliations:** a Industrial Engineering and Operations Research Department, Columbia University, 500 W. 120th Street, New York, NY, 10027, USA; b Department of Electrical Engineering, College of Engineering, Taif University, Taif, 21944, Saudi Arabia; c Department of Mathematics and Computer Science, Transilvania University of Brasov, 29 Eroilor Boulevard, 500036, Brasov, Romania; d Department of Mathematica, Politehnica University of Timișoara, 300006, Timisoara, Romania; e Department of Mathematics, Faculty of Science, Jazan University, Jazan, 45142, Saudi Arabia

**Keywords:** Fuzzy-number, $L_{P}$space over fuzzy domain, Hölder-like inequality, Minkowski's-like inequality, Beckenbach's-like inequality over fuzzy domain, Differentiable convex like mapping, Fuzzy special means

## Abstract

It is widely recognized that fuzzy number theory relies on the characteristic function. However, within the fuzzy realm, the characteristic function transforms into a membership function contingent upon the interval [0,1]. This implies that real numbers and intervals represent exceptional cases of fuzzy numbers. By considering this approach, this paper introduces a new $L_{P}$ space and novel refinements for integral variations of Hölder's inequality which is known as Hölder's-like inequality over fuzzy domain. Numerous prevailing inequalities associated with Hölder's-like inequality can be enhanced through the newly acquired inequalities, as demonstrated through an application. By using newly defined special means, some new versions of integral inequalities have obtained where differentiable mappings are real-valued convex-like (or convex fuzzy) mappings Lastly, nontrivial numerical examples are also included to validate the accuracy of the presented inequalities as they vary with the parameter ꙍ.

## 1 Introduction

The renowned Young inequality for two scalar values is the t-weighted arithmetic–geometric mean inequality. This inequality stipulates that if ⱷ and y are both positive and ɤ is within the interval [0,1], then:

(1) $${ⱷ}^{ɤ}y^{1-ɤ}\leq ɤⱷ+\left(1-ɤ\right)y\text{,}$$

with equality if and only if ⱷ=y. Let p,q>⟩ with $\frac{1}{p}+\frac{1}{q}=1$. Inequality (1), can be written as

(2) $$ⱷy\leq \frac{{ⱷ}^{p}}{p}+\frac{y^{q}}{q}$$

where, ⱷ and y are both positive real numbers.

In this formulation, inequality (2) was employed to establish the renowned Hölder inequality, which stands as one of the fundamental inequalities in analysis. It has broad applications in both pure and applied mathematics and serves as a crucial tool in addressing numerous problems across social, cultural, and natural sciences.

Theorem 1 **(**Hölder-like inequality [1]) Suppose p>1 and $\frac{1}{p}+\frac{1}{q}=1$. If Υ and φ are two real functions defined on [τ,ς] such that ${\left|\Upsilon \right|}^{p}$ and ${\left|\varphi \right|}^{q}$ are integrable functions on [τ,ς], then,

(3) $$\int_{\tau }^{\varsigma }\left|\Upsilon \left(ⱷ\right)\varphi \left(ⱷ\right)\right|dⱷ\leq {\left[\int_{\tau }^{\varsigma }{\left|\Upsilon \left(ⱷ\right)\right|}^{p}dⱷ\right]}^{\frac{1}{p}}+{\left[\int_{\tau }^{\varsigma }{\left|\varphi \left(ⱷ\right)\right|}^{p}dⱷ\right]}^{\frac{1}{p}}$$

with equality if and only if Υ and φ are proportional.

Indeed, Qiang and Hu [2], Tian et al. [3,4] and Wu [5] have been thoroughly explored and applied the Hölder’s inequality in various contexts by numerous researchers. Several generalizations and refinements have been achieved up to this point. Please see, for example [[2], [3], [4], [5]], and the references therein. In this paper, using a straightforward proof method, we establish some new refinements for integral forms of Hölder’s-like inequality over fuzzy domain. For further study, see [[6], [7], [8], [9], [10]] and the references therein.

On the other hand, exploring how mathematical integration principles adapt to ambiguous regions within fuzzy domains is intriguing. Sugeno initially introduced the theory of fuzzy measures and fuzzy integrals in [11]. Developing various types of integral inequalities is a current focus. Recently, numerous valuable studies have been conducted based on different non-additive integrals, including the Sugeno integral [12,13], generalized Sugeno integral [14], pseudo integral [15,16], Choquet integral [17], and others. Set-valued functions [18], serving as a generalization of single-valued functions, have become increasingly important both theoretically and practically. They have become essential tools for addressing problems in various fields, particularly in mathematical economics, such as individual demand, mean demand, competitive equilibrium, and coalition production economies, see [[19], [20], [21], [22], [23], [24], [25], [26], [27]]. It is noteworthy to mention the work by Khan et al. [[28], [29], [30]], which introduced the concept of fuzzy convex inequalities and was one of the most influential publications published in the past year. The idea itself is a vast area that can be studied in further detail. Recently, Khan et al. [31,32] introduced new versions of fuzzy integral inequalities via fuzzy fractional integrals as well as establish relationship between up and down fuzzy relation and inclusion relation. Moreover, some very interested examples also give to support the validity of the results. For further details on this topic, refer to the cited study. For more information, related to fuzzy theory, see [[33], [34], [35], [36], [37], [38], [39], [40], [41]] and the references therein.

Khastan and Rodríguez-López [42] recently introduced real-valued functions across the fuzzy domain and explored specific properties of such functions over fuzziness using Lebesgue measures. After that Khan and Guirao [43] extended this version of integral to fractional integral that integrals are known as Riemann-Liouville fractional-like integrals over fuzzy domain. Moreover, the properties of convex-like functions over fuzzy domain are discussed, see [44,45]. For more information, related to fuzzy inequalities, se [[46], [47], [48], [49]] and the references therein.

Inspired by ongoing research work and special by [42,43], some applications of real-valued functions over fuzzy domain are provided. In this research, after recalling basic concepts and results, a new version of Hölder’s-like inequality over fuzzy domain. Some related inequalities are proved like Minkowski’s-like inequality, Beckenbach’s-like inequality over fuzzy domain, as applications of Hölder-like inequality as well as verified with the support of nontrivial example. Modified version of following means will be used in Section 4:

The positive real numbers ɤ,θ, ɤ≠θ have well-known means in the literature:

$$\begin{array}{rrrrr} A\left(ɤ,\theta \right) & =\frac{ɤ+\theta }{2}\text{,} & & & \;\text{arithmetic}\;\text{mean},\\ G\left(ɤ,\theta \right) & =\sqrt{ɤ\theta }\text{,} & & & \;\text{geometric}\;\text{mean},\\ L\left(ɤ,\theta \right) & =\frac{\theta -ɤ}{\ln\;\theta -\ln\;ɤ}\text{,} & & & \;\text{logarithmic}\;\text{mean},\\ I\left(ɤ,\theta \right) & =\frac{1}{e}{\left(\frac{\theta^{\theta }}{{ɤ}^{ɤ}}\right)}^{1/\left(\theta -ɤ\right)}\text{,} & & & \;\text{identric}\;\text{mean},\\ L_{p}\left(ɤ,\theta \right) & ={\left[\frac{\theta^{p+1}-{ɤ}^{p+1}}{\left(p+1\right)\left(\theta -ɤ\right)}\right]}^{1/p}\text{,} & & & \;\text{generalized}\;\log‐\text{mean},p\neq -1,0 \end{array}$$

## 2 Preliminaries

Firstly, we offer the ideas and concepts needed for the follow-up, see Refs. [[40], [41], [42], [43], [44], [45], [46], [47], [48], [49]]. From Section 3, we offer the primary findings of the paper to guarantee its completion. We begin by defining a fuzzy set in such a way that.

Definition 1 [35] A fuzzy subset T of R is characterized by a mapping $\tilde{ʊ}:R\to \left[0,1\right]$ known as the membership mapping of T, denoted as $\tilde{Ʊ}:R\to \left[0,1\right]$. Hence, for further investigation, we adopt this notation. We designate E to represent the set of all fuzzy subsets of R.

In [37], Goetschel and Voxman introduced the main idea of fuzzy numbers as follows:

Let $\tilde{Ʊ}\in E$. Then, $\tilde{Ʊ}$ is recognized as a fuzzy number or fuzzy interval if it satisfies the following properties:

(1) $\tilde{Ʊ}$ should be normal if there exists ⱷ∈R and $\tilde{Ʊ}\left(ⱷ\right)=1\text{;}$.
(2) $\tilde{Ʊ}$ should be upper semi-continuous on R if for given ⱷ∈R, there exist ε>0 there exist δ>0 such that $\tilde{Ʊ}\left(ⱷ\right)-\tilde{Ʊ}\left(y\right)<\varepsilon$ for all y∈R with |ⱷ−y|<δ;.
(3) $\tilde{Ʊ}$ should be fuzzy convex, meaning $\tilde{Ʊ}\left(\left(1-ɤ\right)ⱷ+ɤy\right)\geq \min\left(\tilde{Ʊ}\left(ⱷ\right),\tilde{Ʊ}\left(y\right)\right)\text{,}$ for all ⱷ,y∈R, and ɤ∈[0,1].
(4) $\tilde{Ʊ}$ should be compactly supported, i.e., $\text{cl}\left\{ⱷ\in R|\;\tilde{Ʊ}\left(ⱷ\right)>0\right\}$ is compact.

We designate $E_{C}$ to represent the set of all fuzzy numbers of R.

Definition 2 [35] Given $\tilde{Ʊ}\in E_{C}$, the level sets or cut sets are defined as ${\left[\tilde{Ʊ}\right]}^{ꙍ}=\left\{ⱷ\in R|\;\tilde{Ʊ}\left(ⱷ\right)\geq ꙍ\right\}$ for all ꙍ∈[0,1].

From these definitions, we have

(4) $${\left[\tilde{Ʊ}\right]}^{ꙍ}=\left[ʌ\left(ꙍ\right),ʋ\left(ꙍ\right)\right]\text{,}$$

where

ʌ(ꙍ)=inf{ⱷ∈R|Ʊ(ⱷ)≥ꙍ},

ʋ(ꙍ)=sup{ⱷ∈R|Ʊ(ⱷ)≥ꙍ}.

Remark 1 [36] For each interval $\left[\tau ,\varsigma \right]\in X_{C}$, there characteristic function $\tilde{\left[\tau ,\varsigma \right]}:R\to \left[0,1\right]$ defined by

(5) $$\tilde{\left[\tau ,\varsigma \right]}\left(ⱷ\right)=\left\{ \begin{array}{c} 1\;ⱷ\in \left[\tau ,\varsigma \right]\\ 0\;\text{otherwise}, \end{array}\right.$$

So, in a way, we can consider that fuzzy numbers extend the set of closed intervals of real numbers, i.e., $X_{C}\subseteq E_{C}$, and consequently $R\subseteq E_{C}$ as well, since degenerated intervals can be interpreted as real numbers. Instead of representing $\tilde{\left[\varsigma ,\varsigma \right]}$, we simply use $\tilde{\varsigma }$. A fuzzy number $\tilde{\varsigma }$ is referred to as a crisp number or fuzzy singleton, as discussed in Ref. [36].

Recalling the concepts commonly found in the literature, if $\tilde{Ʊ},\tilde{\Pi }\in E_{C}$ and ꙍ∈R, then, for every ꙍ∈[0,1], the arithmetic operations are defined as follows, see Ref. [34]:

(6) $${\left[\tilde{Ʊ}⊕\tilde{\Pi }\right]}^{ꙍ}={\left[\tilde{Ʊ}\right]}^{ꙍ}+{\left[\tilde{\Pi }\right]}^{ꙍ}\text{,}$$

(7) $${\left[\tilde{Ʊ}⊗\tilde{\Pi }\right]}^{ꙍ}={\left[\tilde{Ʊ}\right]}^{ꙍ}\times {\left[\;\tilde{\Pi }\right]}^{ꙍ}\text{,}$$

(8) $${\left[ɤ⊙\tilde{Ʊ}\right]}^{ꙍ}=ɤ.{\left[\tilde{Ʊ}\right]}^{ꙍ}\text{.}$$

Theorem 2 [35] The space $E_{C}$ dealing with a supremum metric, i.e., for $\tilde{Ʊ},\tilde{\Pi }\in E_{C}$

(9) $$d_{\infty }\left(\tilde{Ʊ},\tilde{\Pi }\right)=\underset{0\leq ꙍ\leq 1}{\sup}d_{H}\left({\left[\tilde{Ʊ}\right]}^{ꙍ},{\left[\tilde{\Pi }\right]}^{ꙍ}\right)\text{,}$$

is a complete metric space, where H denotes the well-known Hausdorff metric on space of intervals.

Now we recall some the concept of integral over fuzzy domain, where the integrable mappings are real-valued mappings over fuzzy domain.

Definition 3 [42] If $\tilde{Ʊ}\in E_{C}$, and $\Upsilon :\tilde{Ʊ}\to R$ is measurable on ${\left[\tilde{Ʊ}\right]}^{0}$ (and hence on every ${\left[\tilde{Ʊ}\right]}^{ꙍ}$, for all ꙍ∈[0,1]), then we define

(10) $$\left(\int_{\tilde{Ʊ}}\Upsilon \right)\left(ꙍ\right)=\int_{{\left[\tilde{Ʊ}\right]}^{ꙍ}}\Upsilon \left(ⱷ\right)dⱷ\text{,}$$

where the integral on the right-hand side is calculated in the sense of Lebesgue. We say the Υ is integrable over the fuzzy domain; if the integral $\int_{{\left[\tilde{Ʊ}\right]}^{0}}\Upsilon \left(ⱷ\right)dⱷ$ is finite. In that case, mapping is defined as

where the integral on the right-hand side is computed according to Lebesgue integration. We denote that Υ is integrable over the fuzzy domain if the integral $\int_{{\left[\tilde{Ʊ}\right]}^{0}}\Upsilon \left(ⱷ\right)dⱷ$ is finite. In such instances, the mapping is defined as:

$$\int_{\tilde{Ʊ}}\Upsilon :\left[0,1\right]\;\to \;R$$

$$ꙍ\;\to \;\left(\int_{\tilde{Ʊ}}\Upsilon \right)\left(ꙍ\right)=\int_{{\left[\tilde{Ʊ}\right]}^{ꙍ}}\Upsilon \left(ⱷ\right)dⱷ\text{.}$$

Remark 2 By employing Remark 1, we derive the traditional definition of the integral, applicable to real-valued functions that are integrable.

Definition 4 [43] A convex-like real-valued mapping $\Upsilon :\tilde{Ʊ}\to R$ is defined by

(11) Υ(ʎⱷ+(1−ʎ)s)≤ʎΥ(ⱷ)+(1−ʎ)Υ(s),

for all $ⱷ,s\in {\left[\tilde{Ʊ}\right]}^{ꙍ},ʎ\in \left[0,1\right]\text{.}$ Υ is termed as concave-like real-valued mapping over $\tilde{Ʊ}$ if the inequality in (11) is reversed. If Υ is convex-like and concave-like over $\tilde{Ʊ}$, then Υ is affine.

Definition 5 [43] Let $\tilde{Ʊ}\in E_{C}$, and Υ:[τ,ς]⊆R→R is said to be differentiable on ${\left[\tilde{Ʊ}\right]}^{0}\subseteq \left[\tau ,\varsigma \right]$ (and hence for each ${\left[\tilde{Ʊ}\right]}^{ꙍ}$, for all ꙍ∈[0,1]), and ${ʎ}_{0}\in {\left[\tilde{Ʊ}\right]}^{0}$. We define derivative of Υ, $\Upsilon^{\prime }\left({ʎ}_{0}\right)\in R$ (provided it exists) as

(12) $$\left({\Upsilon^{\prime }}_{{ʎ}_{0}\in \tilde{Ʊ}}\right)\left(ꙍ\right)=\lim_{h\to 0^{-}}\frac{\Upsilon \left({ʎ}_{0}+h\right)-\Upsilon \left({ʎ}_{0}\right)}{h}={\Upsilon^{\prime }}_{{ʎ}_{0}\in {\left[\tilde{Ʊ}\right]}^{ꙍ}}\left({ʎ}_{0}\right)\text{.}$$

We call ${\Upsilon^{\prime }}_{{ʎ}_{0}\in {\left[\tilde{Ʊ}\right]}^{ꙍ}}\left({ʎ}_{0}\right)$ the derivative of Υ at ${ʎ}_{0}\in {\left[\tilde{Ʊ}\right]}^{ꙍ}$. Also, we define the left derivative Υ′−(ʎ0)∈R (provided it exists) as

(13) (Υ′ʎ0∈Ʊ˜)(ꙍ)=limh→0−Υ(ʎ0+h)−Υ(ʎ0)h=Υ′−ʎ0∈[Ʊ˜]ꙍ(ʎ0)−.

and the right derivative Υ′+(ʎ0)∈R (provided it exists) as

(14) (Υ′ʎ0∈Ʊ˜)(ꙍ)=limh→0+Υ(ʎ0+h)−Υ(ʎ0)h=Υ′+ʎ0∈[Ʊ˜]ꙍ(ʎ0)+.

We say that Υ is differentiable on $\left[\tilde{Ʊ}\right]$ if it is differentiable at each fuzzy point on $\left[\tilde{Ʊ}\right]$. At the end points of $\left[\tilde{Ʊ}\right]$, we only consider the one sided derivative.

## 3 $L_{P}$ space over fuzzy domain

$L_{P}$ space: A measurable mapping $\Upsilon :\tilde{Ʊ}\to R$ defined on fuzzy number $\tilde{Ʊ}$ is said to be $p^{\text{th}}$ power sum able, where p≥1, if

(15) $$ꙍ\;\to \;\left(\int_{\tilde{Ʊ}}{\left|\Upsilon \right|}^{p}\right)\left(ꙍ\right)=\int_{{\left[\tilde{Ʊ}\right]}^{ꙍ}}{\left|\Upsilon \left(ⱷ\right)\right|}^{p}dⱷ<\infty \text{.}$$

then, $L_{P}$ space is denoted and defined as

$$L_{P}\left[\tilde{Ʊ}\right]=\left\{\Upsilon |\Upsilon :\tilde{Ʊ}\to R\;\text{is}\;\text{measurable}\;\text{on}{\left[\tilde{Ʊ}\right]}^{ꙍ}\text{and}\;\int_{{\left[\tilde{Ʊ}\right]}^{ꙍ}}{\left|\Upsilon \left(ⱷ\right)\right|}^{p}dⱷ<\infty \right\}\text{.}$$

For p=∞, there exist positive W<∞ such that |Υ(ⱷ)|<W.

Remark 3 Utilizing Remark 1 and Remark 2, we derive the classical $L_{P}\tilde{\left[\tau ,\varsigma \right]}$ space.

Particular Cases.

Considering the triangular fuzzy numbers (T· F· N s) $\tilde{Ʊ}=\left(s;\;ᴫ,ʋ\right)$, where s∈R, and ᴫ,ʋ∈R, thus

$$\tilde{Ʊ}\left(ⱷ\right)=\left\{ \begin{array}{c} \frac{ʊ-s+ᴫ}{ᴫ},ʊ\in \left[s-ᴫ,s\right]\\ \frac{\;s+\mu -ʊ}{ʋ},ʊ\in (s,s+\mu ]\\ 0,\text{otherwise}.\; \end{array}\right.$$

**Fig. 1:**
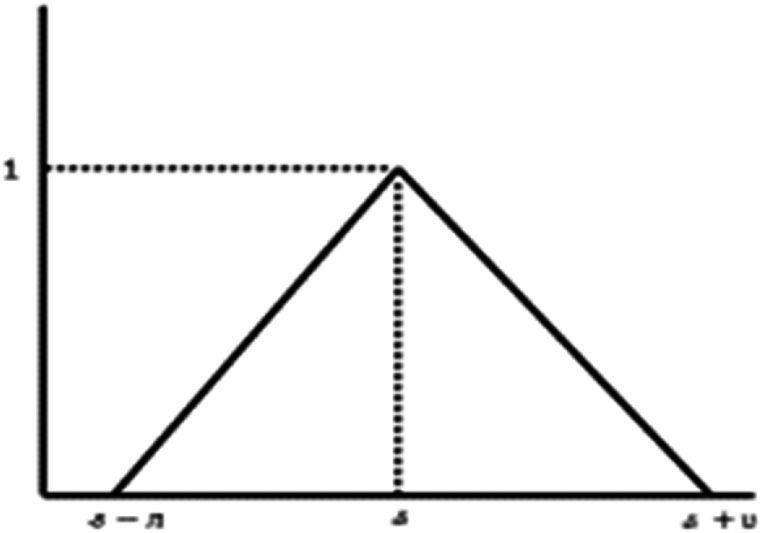
Triangular fuzzy number.

Following is the geometric representation of T· F· N s:

whose parametrized form is ${\left[\tilde{Ʊ}\right]}^{ꙍ}=\left[s-ᴫ\left(1-ꙍ\right),s+\mu \left(1-ꙍ\right)\right]$, for all ꙍ∈[0,1]. Then (see Fig. 1),

$$L_{P}\left[\tilde{Ʊ}\right]=\left\{\Upsilon |\Upsilon :\left[s-ᴫ\left(1-ꙍ\right),s+\mu \left(1-ꙍ\right)\right]\to R\;\text{is}\;\text{measurable}\;\text{on}\left[s-ᴫ\left(1-ꙍ\right),s+\mu \left(1-ꙍ\right)\right]\;\text{and}\;\int_{\left[s-ᴫ\left(1-ꙍ\right),s+\mu \left(1-ꙍ\right)\right]}{\left|\Upsilon \left(ⱷ\right)\right|}^{p}dⱷ<\infty ,\text{for}\;\text{all}\;ꙍ\in \left[0,1\right]\right\}\text{.}$$

On the flip side, considering the trapezoidal fuzzy numbers $\tilde{Ʊ}=\left(s,t;\;ᴫ,ʋ\right)$, where s,t∈R, and ᴫ,ʋ∈R, thus

$$\tilde{Ʊ}\left(ⱷ\right)=\left\{ \begin{array}{c} 1,ʊ\in \left[s,t\right]\\ \frac{ʊ-s+ᴫ}{ᴫ},ʊ\in \left[s-ᴫ,s\right]\\ \frac{\;t+\mu -ʊ}{ʋ},ʊ\in \left[t,t+\mu \right]\\ 0,\text{otherwise}, \end{array}\right.$$

**Fig. 2:**
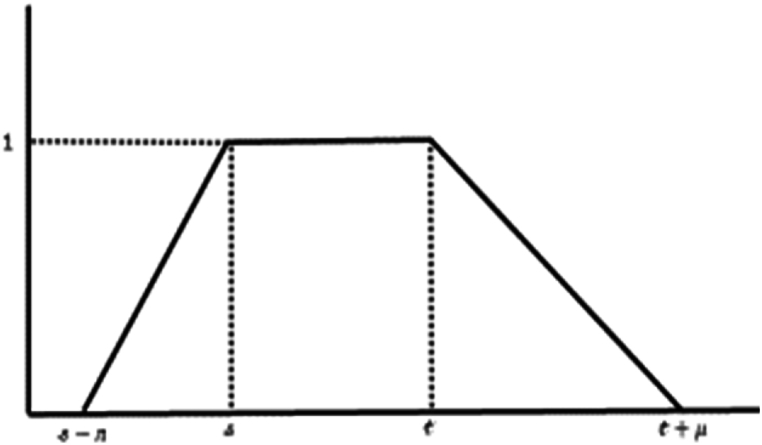
Trapezoidal fuzzy number.

Following is the geometric representation of trapezoidal fuzzy numbers T· F· N:

whose parametrized form is ${\left[\tilde{Ʊ}\right]}^{ꙍ}=\left[s-ᴫ\left(1-ꙍ\right),t+\mu \left(1-ꙍ\right)\right]$, for all ꙍ∈[0,1]. Then (see Fig. 2)

$$L_{P}\left[\tilde{Ʊ}\right]=\left\{\Upsilon |\Upsilon :\left[s-ᴫ\left(1-ꙍ\right),t+\mu \left(1-ꙍ\right)\right]\to R\;\text{is}\;\text{measurable}\;\text{on}\left[\left[s-ᴫ\left(1-ꙍ\right),t+\mu \left(1-ꙍ\right)\right]\right]\;\text{and}\;\int_{\left[\left[s-ᴫ\left(1-ꙍ\right),t+\mu \left(1-ꙍ\right)\right]\right]}{\left|\Upsilon \left(ⱷ\right)\right|}^{p}dⱷ<\infty ,\text{for}\;\text{all}\;ꙍ\in \left[0,1\right]\right\}\text{.}$$

Remark 4 It is commonly known that when t=s, a trapezoidal fuzzy number transforms into a triangular number. Consequently, integrals over trapezoidal fuzzy numbers simplify to integrals over T· F· N s.

All the preceding preliminary concepts are valuable for examining the forthcoming main findings since the focus is on computing the Hölder's-like Inequality within the fuzzy region $\tilde{Ʊ}\in E_{C}$, see Refs. [[4], [5], [6], [7], [8], [9], [10]].

## 4 Hölder like inequality

In this paper, utilizing a straightforward proof method, we acquire following new versions for integral forms of Hölder's inequality, see Refs. [[11], [12], [13], [14], [15], [16], [17], [18], [19], [20]].

Theorem 3 **(**Hölder like inequality) Suppose p>1 and $\frac{1}{p}+\frac{1}{q}=1$. If Υ and φ are two real functions defined on $\tilde{Ʊ}$ such that ${\left|\Upsilon \right|}^{p}$ and ${\left|\varphi \right|}^{q}$ are integrable functions on $\tilde{Ʊ}$, then, for each ꙍ∈[0,1]

(16) $$\left(\int_{\tilde{Ʊ}}\left|\Upsilon \varphi \right|\right)\left(ꙍ\right)\leq {\left[\left(\int_{\tilde{Ʊ}}{\left|\Upsilon \right|}^{p}\right)\left(ꙍ\right)\right]}^{\frac{1}{p}}{\left[\left(\int_{\tilde{Ʊ}}{\left|\varphi \right|}^{q}\right)\left(ꙍ\right)\right]}^{\frac{1}{q}}\text{,}$$

with equality if and only if Υ and φ are proportional.

**Proof**. Since for each ꙍ∈[0,1], we have

$$\left(\int_{\tilde{Ʊ}}\left|\Upsilon \left(ⱷ\right)\varphi \left(ⱷ\right)\right|\right)\left(ꙍ\right)=\int_{{\left[\tilde{Ʊ}\right]}^{ꙍ}}\left|\Upsilon \left(ⱷ\right)\varphi \left(ⱷ\right)\right|dⱷ\text{.}$$

Since ${\left[\tilde{Ʊ}\right]}^{0}=\left[ʌ\left(0\right),ʋ\left(0\right)\right]$ (and hence on every ${\left[\tilde{Ʊ}\right]}^{ꙍ}$, for all ꙍ∈[0,1])

$\int_{{\left[\tilde{Ʊ}\right]}^{0}}\left|\Upsilon \left(ⱷ\right)\varphi \left(ⱷ\right)\right|dⱷ=\int_{ʌ\left(0\right)}^{ʋ\left(0\right)}\left|\Upsilon \left(ⱷ\right)\varphi \left(ⱷ\right)\right|dⱷ$, on every ${\left[\tilde{Ʊ}\right]}^{ꙍ}$, for all ꙍ∈[0,1].

Note that, If $\eta ={\left[\int_{ʌ\left(0\right)}^{ʋ\left(0\right)}{\left|\Upsilon \left(ⱷ\right)\right|}^{p}dⱷ\right]}^{\frac{1}{q}}=0,\text{and}\;\xi ={\left[\int_{ʌ\left(0\right)}^{ʋ\left(0\right)}{\left|\varphi \left(ⱷ\right)\right|}^{q}dⱷ\right]}^{\frac{1}{q}}=0$, it is obvious that equality will holds because functions Υ and φ are measurable on ${\left[\tilde{Ʊ}\right]}^{ꙍ}$.

Considering $\eta ={\left[\int_{ʌ\left(0\right)}^{ʋ\left(0\right)}{\left|\Upsilon \left(ⱷ\right)\right|}^{p}dⱷ\right]}^{\frac{1}{q}}\neq 0,\text{and}\;\xi ={\left[\int_{ʌ\left(0\right)}^{ʋ\left(0\right)}{\left|\varphi \left(ⱷ\right)\right|}^{q}dⱷ\right]}^{\frac{1}{q}}\neq 0$ (and hence on every ${\left[\tilde{Ʊ}\right]}^{ꙍ}$, for all ꙍ∈[0,1]).

Case I. Considering $u=\frac{\left|\Upsilon \left(ⱷ\right)\right|}{\eta },v=\frac{\left|\varphi \left(ⱷ\right)\right|}{\xi }$, Then, by using Auxiliary inequality (2) we have

$$\frac{\left|\Upsilon \left(ⱷ\right)\right|\left|\varphi \left(ⱷ\right)\right|}{\eta \xi }\leq \frac{{\left|\Upsilon \left(ⱷ\right)\right|}^{p}}{p\eta^{p}}+\frac{{\left|\varphi \right|}^{q}}{q\xi^{q}}\text{.}$$

Considering integration over ${\left[\tilde{Ʊ}\right]}^{0}=\left[ʌ\left(0\right),ʋ\left(0\right)\right]$ (and hence on every ${\left[\tilde{Ʊ}\right]}^{ꙍ}$, for all ꙍ∈[0,1]) with respect to ⱷ, we have

$$\frac{1}{\eta \xi }\int_{ʌ\left(0\right)}^{ʋ\left(0\right)}\left|\Upsilon \left(ⱷ\right)\varphi \left(ⱷ\right)\right|dⱷ\leq \frac{1}{p\eta^{p}}\int_{ʌ\left(0\right)}^{ʋ\left(0\right)}{\left|\Upsilon \left(ⱷ\right)\right|}^{p}dⱷ+\frac{1}{q\xi^{q}}\int_{ʌ\left(0\right)}^{ʋ\left(0\right)}{\left|\varphi \left(ⱷ\right)\right|}^{q}dⱷ\text{,}$$

(and hence on every ${\left[\tilde{Ʊ}\right]}^{ꙍ}$, for all ꙍ∈[0,1]), which implies that

$$\frac{1}{\eta \xi }\int_{ʌ\left(0\right)}^{ʋ\left(0\right)}\left|\Upsilon \left(ⱷ\right)\varphi \left(ⱷ\right)\right|dⱷ\leq \frac{1}{p\eta^{p}}\left(\eta^{p}\right)+\frac{1}{q\xi^{q}}\left(\xi^{q}\right)\text{,}$$

$=\frac{1}{p}+\frac{1}{q}=1$, (and hence on every ${\left[\tilde{Ʊ}\right]}^{ꙍ}$, for all ꙍ∈[0,1]).

Then,

$$\int_{ʌ\left(0\right)}^{ʋ\left(0\right)}\left|\Upsilon \left(ⱷ\right)\varphi \left(ⱷ\right)\right|dⱷ\leq {\left[\int_{ʌ\left(0\right)}^{ʋ\left(0\right)}{\left|\Upsilon \left(ⱷ\right)\right|}^{p}dⱷ\right]}^{\frac{1}{p}}{\left[\int_{ʌ\left(0\right)}^{ʋ\left(0\right)}{\left|\varphi \left(ⱷ\right)\right|}^{q}dⱷ\right]}^{\frac{1}{q}}\text{,}$$

(and hence on every ${\left[\tilde{Ʊ}\right]}^{ꙍ}$, for all ꙍ∈[0,1]),which implies that $\left(\int_{\tilde{Ʊ}}\left|\Upsilon \varphi \right|\right)\left(ꙍ\right)\leq {\left[\left(\int_{\tilde{Ʊ}}{\left|\Upsilon \right|}^{p}\right)\left(ꙍ\right)\right]}^{\frac{1}{p}}{\left[\left(\int_{\tilde{Ʊ}}{\left|\varphi \right|}^{q}\right)\left(ꙍ\right)\right]}^{\frac{1}{q}}\text{,}$.

For each ꙍ∈[0,1].

Particular Cases

Here, we explore several exceptional cases that hinge on both triangular and trapezoidal fuzzy numbers.

Firstly, considering T· F· N such that

$${\left[\tilde{Ʊ}\right]}^{ꙍ}=\left[s-ᴫ\left(1-ꙍ\right),s+\mu \left(1-ꙍ\right)\right]\text{,}$$

then inequality (16) simplifies to the Hölder like inequality over T· F· N $\tilde{Ʊ}$ such that

(17) $$\int_{s-ᴫ\left(1-ꙍ\right)}^{s+\mu \left(1-ꙍ\right)}\left|\Upsilon \left(ⱷ\right)\varphi \left(ⱷ\right)\right|dⱷ\leq {\left[\int_{s-ᴫ\left(1-ꙍ\right)}^{s+\mu \left(1-ꙍ\right)}{\left|\Upsilon \left(ⱷ\right)\right|}^{p}dⱷ\right]}^{\frac{1}{p}}{\left[\int_{s-ᴫ\left(1-ꙍ\right)}^{s+\mu \left(1-ꙍ\right)}{\left|\varphi \left(ⱷ\right)\right|}^{q}dⱷ\right]}^{\frac{1}{q}}$$

Secondly, considering trapezoidal fuzzy number such that

$${\left[\tilde{Ʊ}\right]}^{ꙍ}=\left[s-ᴫ\left(1-ꙍ\right),t+\mu \left(1-ꙍ\right)\right]\text{,}$$

then inequality (16) simplifies to the Hölder like inequality over trapezoidal fuzzy number $\tilde{Ʊ}$ such that

(18) $$\int_{s-ᴫ\left(1-ꙍ\right)}^{t+\mu \left(1-ꙍ\right)}\left|\Upsilon \left(ⱷ\right)\varphi \left(ⱷ\right)\right|dⱷ\leq {\left[\int_{s-ᴫ\left(1-ꙍ\right)}^{t+\mu \left(1-ꙍ\right)}{\left|\Upsilon \left(ⱷ\right)\right|}^{p}dⱷ\right]}^{\frac{1}{p}}{\left[\int_{s-ᴫ\left(1-ꙍ\right)}^{t+\mu \left(1-ꙍ\right)}{\left|\varphi \left(ⱷ\right)\right|}^{q}dⱷ\right]}^{\frac{1}{q}}$$

Note that, if t=s, then both inequalities (17) and (18) coincide.

Remark 5 If $\tilde{Ʊ}=\tilde{\left[\tau ,\varsigma \right]}$, then from (16), we get classical Hölder’s-like inequality (3) for real-valued mappings.

Applications of Hölder’s-like inequality under convex-like real-valued mapping:

When we obtain $\left|\Upsilon \right|\left|\varphi \right|=\left({\left|\Upsilon \right|}^{\frac{1}{p}}\right)\left({\left|\Upsilon \right|}^{\frac{1}{q}}\left|\varphi \right|\right)$, as a straightforward outcome of the Hölder Inequality, we have the Hölder’s power-mean-like integral inequality that follows:

Theorem 4 Suppose p>1. If Υ and φ are two real functions defined on fuzzy number $\tilde{Ʊ}$ such that |Υ| and ${\left|\Upsilon \right|}^{p}\left|\varphi \right|$ are integrable functions on $\tilde{Ʊ}$, then:

(19) $$\left(\int_{\tilde{Ʊ}}\left|\Upsilon \varphi \right|\right)\left(ꙍ\right)\leq {\left[\left(\int_{\tilde{Ʊ}}{\left|\Upsilon \right|}^{p}\right)\left(ꙍ\right)\right]}^{\frac{1}{p}}{\left[\left(\int_{\tilde{Ʊ}}\left|\Upsilon \right|{\left|\varphi \right|}^{p}\right)\left(ꙍ\right)\right]}^{1-\frac{1}{p}}\text{,}$$

**Proof.** By using same arguments like Theorem 3, it can be proved.

If p=2=q, then we attain the following outcome:

Corollary 1 (Cauchy-Schwarz’s-like inequality) In accordance with the premises of Theorem 3, if p=2=q, then, it is evident that

(20) $$\left(\int_{\tilde{Ʊ}}\left|\Upsilon \varphi \right|\right)\left(ꙍ\right)\leq {\left[\left(\int_{\tilde{Ʊ}}{\left|\Upsilon \right|}^{2}\right)\left(ꙍ\right)\right]}^{\frac{1}{2}}{\left[\left(\int_{\tilde{Ʊ}}{\left|\varphi \right|}^{2}\right)\left(ꙍ\right)\right]}^{\frac{1}{2}}\text{,}$$

for each ꙍ∈[0,1].

## 5 Minkowski-like inequality

Theorem 5 (Minkowski’s-like inequality) Suppose p≥1. If Υ and φ are two real functions defined on $\tilde{Ʊ}$ such that ${\left|\Upsilon \right|}^{p}$ and ${\left|\varphi \right|}^{p}$ are integrable functions on $\tilde{Ʊ}$, then, for each ꙍ∈[0,1]

(21) $${\left[\left(\int_{\tilde{Ʊ}}{\left|\Upsilon +\varphi \right|}^{p}\right)\left(ꙍ\right)\right]}^{\frac{1}{p}}\leq {\left[\left(\int_{\tilde{Ʊ}}{\left|\Upsilon \right|}^{p}\right)\left(ꙍ\right)\right]}^{\frac{1}{p}}{+\left[\left(\int_{\tilde{Ʊ}}{\left|\varphi \right|}^{p}\right)\left(ꙍ\right)\right]}^{\frac{1}{p}}\text{,}$$

with equality if and only if Υ and φ are proportional.

If 1>p>0, then

(22) $${\left[\left(\int_{\tilde{Ʊ}}{\left|\Upsilon +\varphi \right|}^{p}\right)\left(ꙍ\right)\right]}^{\frac{1}{p}}\geq {\left[\left(\int_{\tilde{Ʊ}}{\left|\Upsilon \right|}^{p}\right)\left(ꙍ\right)\right]}^{\frac{1}{p}}{+\left[\left(\int_{\tilde{Ʊ}}{\left|\varphi \right|}^{p}\right)\left(ꙍ\right)\right]}^{\frac{1}{p}}\text{,}$$

**Proof. Case I.** Suppose that p=1 and we know that

|Υ(ⱷ)+φ(ⱷ)|≤|Υ(ⱷ)|+|φ(ⱷ)|.

Considering integration on the both side over ${\left[\tilde{Ʊ}\right]}^{0}=\left[ʌ\left(0\right),ʋ\left(0\right)\right]$ (and hence on every ${\left[\tilde{Ʊ}\right]}^{ꙍ}$, for all ꙍ∈[0,1]), we have

$$\int_{ʌ\left(0\right)}^{ʋ\left(0\right)}\left|\Upsilon \left(ⱷ\right)+\varphi \left(ⱷ\right)\right|dⱷ\leq \int_{ʌ\left(0\right)}^{ʋ\left(0\right)}\left|\Upsilon \left(ⱷ\right)\right|dⱷ+\int_{ʌ\left(0\right)}^{ʋ\left(0\right)}\left|\varphi \left(ⱷ\right)\right|dⱷ\text{,}$$

which implies that $\left(\int_{\tilde{Ʊ}}\left|\Upsilon \varphi \right|\right)\left(ꙍ\right)\leq \left(\int_{\tilde{Ʊ}}\left|\Upsilon \right|\right)\left(ꙍ\right)+\left(\int_{\tilde{Ʊ}}\left|\varphi \right|\right)\left(ꙍ\right)\text{.}$.

**Case II. Consider that**p>1 and that p and q are conjugate indices. Then,

$$\int_{ʌ\left(0\right)}^{ʋ\left(0\right)}{\left|\Upsilon \left(ⱷ\right)+\varphi \left(ⱷ\right)\right|}^{p}dⱷ=\int_{ʌ\left(0\right)}^{ʋ\left(0\right)}{\left|\Upsilon \left(ⱷ\right)+\varphi \left(ⱷ\right)\right|\left|\Upsilon \left(ⱷ\right)+\varphi \left(ⱷ\right)\right|}^{p-1}dⱷ$$

$$=\int_{ʌ\left(0\right)}^{ʋ\left(0\right)}{\left(\left|\Upsilon \left(ⱷ\right)\right|+\left|\varphi \left(ⱷ\right)\right|\right)\left|\Upsilon \left(ⱷ\right)+\varphi \left(ⱷ\right)\right|}^{p-1}dⱷ$$

$$=\int_{ʌ\left(0\right)}^{ʋ\left(0\right)}\left|\Upsilon \left(ⱷ\right)\right|{\left|\Upsilon \left(ⱷ\right)+\varphi \left(ⱷ\right)\right|}^{p-1}dⱷ+\int_{ʌ\left(0\right)}^{ʋ\left(0\right)}{\left|\varphi \left(ⱷ\right)\right|\left|\Upsilon \left(ⱷ\right)+\varphi \left(ⱷ\right)\right|}^{p-1}dⱷ\text{,}$$

and hence on every ${\left[\tilde{Ʊ}\right]}^{ꙍ}$, for all ꙍ∈[0,1].

By using Hölder like Inequality, we have

$$\int_{ʌ\left(0\right)}^{ʋ\left(0\right)}{\left|\Upsilon \left(ⱷ\right)+\varphi \left(ⱷ\right)\right|}^{p}dⱷ\leq {\left[\int_{ʌ\left(0\right)}^{ʋ\left(0\right)}{\left|\Upsilon \left(ⱷ\right)\right|}^{p}dⱷ\right]}^{\frac{1}{p}}{\left[\int_{ʌ\left(0\right)}^{ʋ\left(0\right)}{\left|\Upsilon \left(ⱷ\right)+\varphi \left(ⱷ\right)\right|}^{\left(p-1\right)q}dⱷ\right]}^{\frac{1}{q}}$$

$$+{\left[\int_{ʌ\left(0\right)}^{ʋ\left(0\right)}{\left|\varphi \left(ⱷ\right)\right|}^{p}dⱷ\right]}^{\frac{1}{p}}{\left[\int_{ʌ\left(0\right)}^{ʋ\left(0\right)}{\left|\Upsilon \left(ⱷ\right)+\varphi \left(ⱷ\right)\right|}^{\left(p-1\right)q}dⱷ\right]}^{\frac{1}{q}}$$

$$=\left({\left[\int_{ʌ\left(0\right)}^{ʋ\left(0\right)}{\left|\Upsilon \left(ⱷ\right)\right|}^{p}dⱷ\right]}^{\frac{1}{p}}+{\left[\int_{ʌ\left(0\right)}^{ʋ\left(0\right)}{\left|\varphi \left(ⱷ\right)\right|}^{p}dⱷ\right]}^{\frac{1}{p}}\right){\left[\int_{ʌ\left(0\right)}^{ʋ\left(0\right)}{\left|\Upsilon \left(ⱷ\right)+\varphi \left(ⱷ\right)\right|}^{\left(p-1\right)q}dⱷ\right]}^{\frac{1}{q}}\text{,}$$

which implies, we have

$$\int_{ʌ\left(0\right)}^{ʋ\left(0\right)}{\left|\Upsilon \left(ⱷ\right)+\varphi \left(ⱷ\right)\right|}^{p}dⱷ\leq \left({\left[\int_{ʌ\left(0\right)}^{ʋ\left(0\right)}{\left|\Upsilon \left(ⱷ\right)\right|}^{p}dⱷ\right]}^{\frac{1}{p}}+{\left[\int_{ʌ\left(0\right)}^{ʋ\left(0\right)}{\left|\varphi \left(ⱷ\right)\right|}^{p}dⱷ\right]}^{\frac{1}{p}}\right){\left[\int_{ʌ\left(0\right)}^{ʋ\left(0\right)}{\left|\Upsilon \left(ⱷ\right)+\varphi \left(ⱷ\right)\right|}^{p}dⱷ\right]}^{\frac{1}{q}}$$

$$\leq \left({\left[\int_{ʌ\left(0\right)}^{ʋ\left(0\right)}{\left|\Upsilon \left(ⱷ\right)\right|}^{p}dⱷ\right]}^{\frac{1}{p}}+{\left[\int_{ʌ\left(0\right)}^{ʋ\left(0\right)}{\left|\varphi \left(ⱷ\right)\right|}^{p}dⱷ\right]}^{\frac{1}{p}}\right){\left[{\left(\int_{ʌ\left(0\right)}^{ʋ\left(0\right)}{\left|\Upsilon \left(ⱷ\right)+\varphi \left(ⱷ\right)\right|}^{p}dⱷ\right)}^{\frac{1}{p}}\right]}^{\frac{p}{q}}\text{.}$$

From above inequality, we have

$${\left({\left(\int_{ʌ\left(0\right)}^{ʋ\left(0\right)}{\left|\Upsilon \left(ⱷ\right)+\varphi \left(ⱷ\right)\right|}^{p}dⱷ\right)}^{\frac{1}{p}}\right)}^{p}{\left[{\left(\int_{ʌ\left(0\right)}^{ʋ\left(0\right)}{\left|\Upsilon \left(ⱷ\right)+\varphi \left(ⱷ\right)\right|}^{p}dⱷ\right)}^{\frac{1}{p}}\right]}^{-\frac{p}{q}}\leq {\left[\int_{ʌ\left(0\right)}^{ʋ\left(0\right)}{\left|\Upsilon \left(ⱷ\right)\right|}^{p}dⱷ\right]}^{\frac{1}{p}}+{\left[\int_{ʌ\left(0\right)}^{ʋ\left(0\right)}{\left|\varphi \left(ⱷ\right)\right|}^{p}dⱷ\right]}^{\frac{1}{p}}\text{,}$$

implies that

$${\left(\int_{ʌ\left(0\right)}^{ʋ\left(0\right)}{\left|\Upsilon \left(ⱷ\right)+\varphi \left(ⱷ\right)\right|}^{p}dⱷ\right)}^{\frac{1}{p}}\leq {\left[\int_{ʌ\left(0\right)}^{ʋ\left(0\right)}{\left|\Upsilon \left(ⱷ\right)\right|}^{p}dⱷ\right]}^{\frac{1}{p}}+{\left[\int_{ʌ\left(0\right)}^{ʋ\left(0\right)}{\left|\varphi \left(ⱷ\right)\right|}^{p}dⱷ\right]}^{\frac{1}{p}}\text{,}$$

and hence on every ${\left[\tilde{Ʊ}\right]}^{ꙍ}$, for all ꙍ∈[0,1].

Hence,

$${\left[\left(\int_{\tilde{Ʊ}}{\left|\Upsilon +\varphi \right|}^{p}\right)\left(ꙍ\right)\right]}^{\frac{1}{p}}\leq {\left[\left(\int_{\tilde{Ʊ}}{\left|\Upsilon \right|}^{p}\right)\left(ꙍ\right)\right]}^{\frac{1}{p}}{+\left[\left(\int_{\tilde{Ʊ}}{\left|\varphi \right|}^{p}\right)\left(ꙍ\right)\right]}^{\frac{1}{p}}\text{.}$$

Particular Cases

Here, we explore several exceptional cases that hinge on both triangular and trapezoidal fuzzy numbers.

Firstly, considering T· F· N such that

$${\left[\tilde{Ʊ}\right]}^{ꙍ}=\left[s-ᴫ\left(1-ꙍ\right),s+\mu \left(1-ꙍ\right)\right]\text{,}$$

then inequalities (21) and (22), simplifies to the Minkowski-like inequalities over T· F· N $\tilde{Ʊ}$ such that

(23) $${\left(\int_{s-ᴫ\left(1-ꙍ\right)}^{s+\mu \left(1-ꙍ\right)}{\left|\Upsilon \left(ⱷ\right)+\varphi \left(ⱷ\right)\right|}^{p}dⱷ\right)}^{\frac{1}{p}}\leq {\left[\int_{s-ᴫ\left(1-ꙍ\right)}^{s+\mu \left(1-ꙍ\right)}{\left|\Upsilon \left(ⱷ\right)\right|}^{p}dⱷ\right]}^{\frac{1}{p}}+{\left[\int_{s-ᴫ\left(1-ꙍ\right)}^{s+\mu \left(1-ꙍ\right)}{\left|\varphi \left(ⱷ\right)\right|}^{p}dⱷ\right]}^{\frac{1}{p}}$$

Secondly, considering trapezoidal fuzzy number such that

$${\left[\tilde{Ʊ}\right]}^{ꙍ}=\left[s-ᴫ\left(1-ꙍ\right),t+\mu \left(1-ꙍ\right)\right]\text{,}$$

then inequalities (21) and (22), simplifies to the Minkowski-like inequalities over trapezoidal fuzzy number $\tilde{Ʊ}$ such that

(24) $${\left(\int_{s-ᴫ\left(1-ꙍ\right)}^{t+\mu \left(1-ꙍ\right)}{\left|\Upsilon \left(ⱷ\right)+\varphi \left(ⱷ\right)\right|}^{p}dⱷ\right)}^{\frac{1}{p}}\leq {\left[\int_{s-ᴫ\left(1-ꙍ\right)}^{t+\mu \left(1-ꙍ\right)}{\left|\Upsilon \left(ⱷ\right)\right|}^{p}dⱷ\right]}^{\frac{1}{p}}+{\left[\int_{s-ᴫ\left(1-ꙍ\right)}^{t+\mu \left(1-ꙍ\right)}{\left|\varphi \left(ⱷ\right)\right|}^{p}dⱷ\right]}^{\frac{1}{p}}\text{.}$$

Note that, if t=s, then both inequalities (23) and (24) coincide.

Remark 6 If $\tilde{Ʊ}=\tilde{\left[\tau ,\varsigma \right]}$, then from (21), (22), we get classical Minkowski’s inequality for real-valued mappings.

## 6 Beckenbach's inequality

Theorem 6 (Beckenbach’s inequality) Suppose 1>p>0. If Υ and φ are two real functions defined on $\tilde{Ʊ}$ and Υ(ⱷ)>0,φ(ⱷ)>0, then

(25) $$\frac{\int_{\tilde{Ʊ}}{\left(\Upsilon +\varphi \right)}^{p+1}\left(ꙍ\right)}{\int_{\tilde{Ʊ}}{\left(\Upsilon +\varphi \right)}^{p}\left(ꙍ\right)}\leq \frac{\int_{\tilde{Ʊ}}{\left(\Upsilon \right)}^{p+1}\left(ꙍ\right)}{\int_{\tilde{Ʊ}}{\left(\Upsilon \right)}^{p}\left(ꙍ\right)}+\frac{\int_{\tilde{Ʊ}}{\left(\varphi \right)}^{p+1}\left(ꙍ\right)}{\int_{\tilde{Ʊ}}{\left(\varphi \right)}^{p}\left(ꙍ\right)}\text{,}$$

with equality if Υ and φ are proportional.

**Proof**. Considering

$$l_{1}={\left[\left(\int_{\tilde{Ʊ}}{\left(\Upsilon \right)}^{p+1}\right)\left(ꙍ\right)\right]}^{\frac{1}{p+1}},l_{2}={\left[\left(\int_{\tilde{Ʊ}}{\left(\varphi \right)}^{p+1}\right)\left(ꙍ\right)\right]}^{\frac{1}{p+1}}$$

and

$$J_{1}={\left[\left(\int_{\tilde{Ʊ}}{\left(\Upsilon \right)}^{p}\right)\left(ꙍ\right)\right]}^{\frac{1}{p}},J_{2}={\left[\left(\int_{\tilde{Ʊ}}{\left(\varphi \right)}^{p}\right)\left(ꙍ\right)\right]}^{\frac{1}{p}}\text{.}$$

Now by using Randon inequality for real number, we have

$$\frac{{l_{1}}^{p+1}}{{J_{1}}^{p}}+\frac{{l_{2}}^{p+1}}{{J_{2}}^{p}}\geq \frac{{\left(l_{1}+l_{2}\right)}^{p+1}}{{\left(J_{1}+J_{2}\right)}^{p}}\text{,}$$

that is to say

(26) $$\frac{\int_{ʌ\left(0\right)}^{ʋ\left(0\right)}{\left(\Upsilon \left(ⱷ\right)\right)}^{p+1}dⱷ}{\int_{ʌ\left(0\right)}^{ʋ\left(0\right)}{\left(\Upsilon \left(ⱷ\right)\right)}^{p}dⱷ}+\frac{\int_{ʌ\left(0\right)}^{ʋ\left(0\right)}{\left(\varphi \left(ⱷ\right)\right)}^{p+1}dⱷ}{\int_{ʌ\left(0\right)}^{ʋ\left(0\right)}{\left(\varphi \left(ⱷ\right)\right)}^{p}dⱷ}\geq \frac{{\left({\left(\int_{ʌ\left(0\right)}^{ʋ\left(0\right)}{\left(\Upsilon \left(ⱷ\right)\right)}^{p+1}dⱷ\right)}^{\frac{1}{p+1}}+{\left(\int_{ʌ\left(0\right)}^{ʋ\left(0\right)}{\left(\varphi \left(ⱷ\right)\right)}^{p+1}dⱷ\right)}^{\frac{1}{p+1}}\right)}^{p+1}}{{\left({\left(\int_{ʌ\left(0\right)}^{ʋ\left(0\right)}{\left(\Upsilon \left(ⱷ\right)\right)}^{p}dⱷ\right)}^{\frac{1}{p}}+{\left(\int_{ʌ\left(0\right)}^{ʋ\left(0\right)}{\left(\varphi \left(ⱷ\right)\right)}^{p}dⱷ\right)}^{\frac{1}{p}}\right)}^{p}}$$

and hence on every ${\left[\tilde{Ʊ}\right]}^{ꙍ}$, for all ꙍ∈[0,1].

Now because 1>p>0, then 2>p+1>1, from (21), (22), we achieve

(27) $${\left[\int_{ʌ\left(0\right)}^{ʋ\left(0\right)}{\left(\Upsilon \left(ⱷ\right)+\varphi \left(ⱷ\right)\right)}^{p+1}dⱷ\right]}^{\frac{1}{p+1}}\leq {\left(\int_{ʌ\left(0\right)}^{ʋ\left(0\right)}{\left(\Upsilon \left(ⱷ\right)\right)}^{p+1}dⱷ\right)}^{\frac{1}{p+1}}+{\left(\int_{ʌ\left(0\right)}^{ʋ\left(0\right)}{\left(\varphi \left(ⱷ\right)\right)}^{p+1}dⱷ\right)}^{\frac{1}{p+1}}\text{,}$$

and

(28) $${\left[\int_{ʌ\left(0\right)}^{ʋ\left(0\right)}{\left(\Upsilon \left(ⱷ\right)+\varphi \left(ⱷ\right)\right)}^{p}dⱷ\right]}^{\frac{1}{p}}\geq {\left(\int_{ʌ\left(0\right)}^{ʋ\left(0\right)}{\left(\Upsilon \left(ⱷ\right)\right)}^{p}dⱷ\right)}^{\frac{1}{p}}+{\left(\int_{ʌ\left(0\right)}^{ʋ\left(0\right)}{\left(\varphi \left(ⱷ\right)\right)}^{p}dⱷ\right)}^{\frac{1}{p}}\text{.}$$

As we know that, if a,b,c>0, then we have

(29) $$a\geq c\Leftrightarrow \frac{a}{b}\geq \frac{c}{b}\text{,}$$

(30) $$b\leq c\Leftrightarrow \frac{a}{b}\geq \frac{a}{c}\text{.}$$

Finally, from (27), (28), (29), (30), we have

(31) $$\frac{{\left({\left(\int_{ʌ\left(0\right)}^{ʋ\left(0\right)}{\left(\Upsilon \left(ⱷ\right)\right)}^{p+1}dⱷ\right)}^{\frac{1}{p+1}}+{\left(\int_{ʌ\left(0\right)}^{ʋ\left(0\right)}{\left(\varphi \left(ⱷ\right)\right)}^{p+1}dⱷ\right)}^{\frac{1}{p+1}}\right)}^{p+1}}{{\left({\left(\int_{ʌ\left(0\right)}^{ʋ\left(0\right)}{\left(\Upsilon \left(ⱷ\right)\right)}^{p}dⱷ\right)}^{\frac{1}{p}}+{\left(\int_{ʌ\left(0\right)}^{ʋ\left(0\right)}{\left(\varphi \left(ⱷ\right)\right)}^{p}dⱷ\right)}^{\frac{1}{p}}\right)}^{p}}\geq \frac{\int_{ʌ\left(0\right)}^{ʋ\left(0\right)}{\left(\Upsilon \left(ⱷ\right)+\varphi \left(ⱷ\right)\right)}^{p+1}dⱷ}{\int_{ʌ\left(0\right)}^{ʋ\left(0\right)}{\left(\Upsilon \left(ⱷ\right)+\varphi \left(ⱷ\right)\right)}^{p}dⱷ}\text{,}$$

and hence on every ${\left[\tilde{Ʊ}\right]}^{ꙍ}$, for all ꙍ∈[0,1]. Hence, from (26), (31), we conclude the required result.

Particular Cases

Here, we explore several exceptional cases that hinge on both triangular and trapezoidal fuzzy numbers.

Firstly, considering T· F· N such that

(32) $${\left[\tilde{Ʊ}\right]}^{ꙍ}=\left[s-ᴫ\left(1-ꙍ\right),s+\mu \left(1-ꙍ\right)\right]\text{,}$$

then inequality (25) simplifies to the Beckenbach-like inequality over T· F· N $\tilde{Ʊ}$ such that

(33) $$\frac{\int_{s-ᴫ\left(1-ꙍ\right)}^{s+\mu \left(1-ꙍ\right)}{\left(\Upsilon \left(ⱷ\right)+\varphi \left(ⱷ\right)\right)}^{p+1}dⱷ}{\int_{s-ᴫ\left(1-ꙍ\right)}^{s+\mu \left(1-ꙍ\right)}{\left(\Upsilon \left(ⱷ\right)+\varphi \left(ⱷ\right)\right)}^{p}dⱷ}\leq \frac{\int_{s-ᴫ\left(1-ꙍ\right)}^{s+\mu \left(1-ꙍ\right)}{\left(\Upsilon \left(ⱷ\right)\right)}^{p+1}dⱷ}{\int_{s-ᴫ\left(1-ꙍ\right)}^{s+\mu \left(1-ꙍ\right)}{\left(\Upsilon \left(ⱷ\right)\right)}^{p}dⱷ}+\frac{\int_{s-ᴫ\left(1-ꙍ\right)}^{s+\mu \left(1-ꙍ\right)}{\left(\varphi \left(ⱷ\right)\right)}^{p+1}dⱷ}{\int_{s-ᴫ\left(1-ꙍ\right)}^{s+\mu \left(1-ꙍ\right)}{\left(\varphi \left(ⱷ\right)\right)}^{p}dⱷ}\text{.}$$

Secondly, considering trapezoidal fuzzy number such that

$${\left[\tilde{Ʊ}\right]}^{ꙍ}=\left[s-ᴫ\left(1-ꙍ\right),t+\mu \left(1-ꙍ\right)\right]\text{,}$$

then inequality (25) simplifies to the Beckenbach-like inequality inequality over trapezoidal fuzzy number $\tilde{Ʊ}$ such that

(34) $$\frac{\int_{s-ᴫ\left(1-ꙍ\right)}^{t+\mu \left(1-ꙍ\right)}{\left(\Upsilon \left(ⱷ\right)+\varphi \left(ⱷ\right)\right)}^{p+1}dⱷ}{\int_{s-ᴫ\left(1-ꙍ\right)}^{t+\mu \left(1-ꙍ\right)}{\left(\Upsilon \left(ⱷ\right)+\varphi \left(ⱷ\right)\right)}^{p}dⱷ}\leq \frac{\int_{s-ᴫ\left(1-ꙍ\right)}^{t+\mu \left(1-ꙍ\right)}{\left(\Upsilon \left(ⱷ\right)\right)}^{p+1}dⱷ}{\int_{s-ᴫ\left(1-ꙍ\right)}^{t+\mu \left(1-ꙍ\right)}{\left(\Upsilon \left(ⱷ\right)\right)}^{p}dⱷ}+\frac{\int_{s-ᴫ\left(1-ꙍ\right)}^{t+\mu \left(1-ꙍ\right)}{\left(\varphi \left(ⱷ\right)\right)}^{p+1}dⱷ}{\int_{s-ᴫ\left(1-ꙍ\right)}^{t+\mu \left(1-ꙍ\right)}{\left(\varphi \left(ⱷ\right)\right)}^{p}dⱷ}$$

Note that, if t=s, then both inequalities (39) and (41) coincide.

Remark 7 If $\tilde{Ʊ}=\tilde{\left[\tau ,\varsigma \right]}$, then from (25), we get following classical Beckenbach’s inequality for real-valued mappings.

Example 1 Consider the trapezoidal fuzzy numbers $\tilde{Ʊ}=\left(1,2;\frac{1}{2},2\right)$, that is

(35) $$\tilde{Ʊ}\left(ⱷ\right)=\left\{ \begin{array}{c} 1,ʊ\in \left[1,2\right]\\ \frac{ʊ-\frac{1}{2}}{ᴫ},ʊ\in \left[1-\frac{1}{2},1\right]\\ \frac{\;4-ʊ}{2},ʊ\in \left[2,2+2\right]\\ 0,\text{otherwise}, \end{array}\right.$$

whose parametrized form is ${\left[\tilde{Ʊ}\right]}^{ꙍ}=\left[1+\frac{1}{2}\left(ꙍ-1\right),2+2\left(1-ꙍ\right)\right]$, for all ꙍ∈[0,1]. Let $p=\frac{1}{2}$, and Υ(ⱷ)=ⱷ and $\varphi \left(ⱷ\right)={ⱷ}^{2}$ be the real-valued mappings on fuzzy domain $\tilde{Ʊ}$.

$$\int_{s-ᴫ\left(1-ꙍ\right)}^{t+\mu \left(1-ꙍ\right)}{\left(\Upsilon \left(ⱷ\right)\right)}^{p+1}dⱷ=\frac{1}{20}\left({8\left(4-2ꙍ\right)}^{\frac{5}{2}}-{\sqrt{2}\left(ꙍ+1\right)}^{\frac{5}{2}}\right)$$

$$\int_{s-ᴫ\left(1-ꙍ\right)}^{t+\mu \left(1-ꙍ\right)}{\left(\Upsilon \left(ⱷ\right)\right)}^{p}dⱷ=\frac{1}{6}\left({4\left(4-2ꙍ\right)}^{\frac{3}{2}}-{\sqrt{2}\left(ꙍ+1\right)}^{\frac{3}{2}}\right)$$

$$\int_{s-ᴫ\left(1-ꙍ\right)}^{t+\mu \left(1-ꙍ\right)}{\left(\varphi \left(ⱷ\right)\right)}^{p+1}dⱷ=\frac{1}{4}\left({16\left(ꙍ-2\right)}^{4}-{\frac{1}{16}\left(ꙍ+1\right)}^{4}\right)$$

$$\int_{s-ᴫ\left(1-ꙍ\right)}^{t+\mu \left(1-ꙍ\right)}{\left(\varphi \left(ⱷ\right)\right)}^{p}dⱷ=\frac{3}{8}\left({5ꙍ}^{2}-22ꙍ+21\right)$$

$$\int_{s-ᴫ\left(1-ꙍ\right)}^{t+\mu \left(1-ꙍ\right)}{\left(\Upsilon \left(ⱷ\right)+\varphi \left(ⱷ\right)\right)}^{p+1}dⱷ=\frac{1}{64}\left\{\sqrt{\left(4-2ꙍ\right)\left(5-2ꙍ\right)}\left(9-4ꙍ\right))\left(16\left(2-ꙍ\right)\left(5-2ꙍ\right)-3\right)-\sqrt{\frac{1+ꙍ}{2}\left(\frac{3+ꙍ}{2}\right)}\left(2+ꙍ\right)\left(4\left(1+ꙍ\right)\left(\frac{3+ꙍ}{2}\right)-3\right)+3\;\ln\left(\frac{\sqrt{4-2ꙍ}+\sqrt{5-2ꙍ}}{\sqrt{\frac{1+ꙍ}{2}}+\sqrt{\frac{3+ꙍ}{2}}}\right)\right\}$$

$$\int_{s-ᴫ\left(1-ꙍ\right)}^{t+\mu \left(1-ꙍ\right)}{\left(\Upsilon \left(ⱷ\right)+\varphi \left(ⱷ\right)\right)}^{p}dⱷ=\frac{1}{4}\left\{\sqrt{\left(4-2ꙍ\right)\left(5-2ꙍ\right)}\left(9-4ꙍ-\frac{\sinh^{-1}\left(\sqrt{4-2ꙍ}\right)}{\sqrt{\left(4-2ꙍ\right)\left(5-2ꙍ\right)}}\right)-\sqrt{\left(\frac{1+ꙍ}{2}\right)\left(\frac{3+ꙍ}{2}\right)}\left(2+ꙍ-\frac{\sinh^{-1}\left(\sqrt{\frac{1+ꙍ}{2}}\right)}{\sqrt{\left(\frac{1+ꙍ}{2}\right)\left(\frac{3+ꙍ}{2}\right)}}\right)\right\}\text{.}$$

Now

$${\left[\int_{s-ᴫ\left(1-ꙍ\right)}^{t+\mu \left(1-ꙍ\right)}{\left(\Upsilon \left(ⱷ\right)\right)}^{p+1}dⱷ\right]}^{\frac{1}{p+1}}={\left(\frac{1}{20}\left({8\left(4-2ꙍ\right)}^{\frac{5}{2}}-{\sqrt{2}\left(ꙍ+1\right)}^{\frac{5}{2}}\right)\right)}^{\frac{2}{3}}$$

$${\left[\int_{s-ᴫ\left(1-ꙍ\right)}^{t+\mu \left(1-ꙍ\right)}{\left(\varphi \left(ⱷ\right)\right)}^{p+1}dⱷ\right]}^{\frac{1}{p+1}}={\left(\frac{1}{4}\left({16\left(ꙍ-2\right)}^{4}-{\frac{1}{16}\left(ꙍ+1\right)}^{4}\right)\right)}^{\frac{2}{3}}$$

$${\left(\int_{s-ᴫ\left(1-ꙍ\right)}^{t+\mu \left(1-ꙍ\right)}{\left(\Upsilon \left(ⱷ\right)+\varphi \left(ⱷ\right)\right)}^{p+1}dⱷ\right)}^{\frac{2}{3}}={\left[\frac{1}{64}\left\{\sqrt{\left(4-2ꙍ\right)\left(5-2ꙍ\right)}\left(9-4ꙍ\right))\left(16\left(2-ꙍ\right)\left(5-2ꙍ\right)-3\right)-\sqrt{\frac{1+ꙍ}{2}\left(\frac{3+ꙍ}{2}\right)}\left(2+ꙍ\right)\left(4\left(1+ꙍ\right)\left(\frac{3+ꙍ}{2}\right)-3\right)+3\;\ln\left(\frac{\sqrt{4-2ꙍ}+\sqrt{5-2ꙍ}}{\sqrt{\frac{1+ꙍ}{2}}+\sqrt{\frac{3+ꙍ}{2}}}\right)\right\}\right]}^{\frac{2}{3}}$$

Then

$${\left[\frac{1}{64}\left\{\sqrt{\left(4-2ꙍ\right)\left(5-2ꙍ\right)}\left(9-4ꙍ\right))\left(16\left(2-ꙍ\right)\left(5-2ꙍ\right)-3\right)-\sqrt{\frac{1+ꙍ}{2}\left(\frac{3+ꙍ}{2}\right)}\left(2+ꙍ\right)\left(4\left(1+ꙍ\right)\left(\frac{3+ꙍ}{2}\right)-3\right)+3\;\ln\left(\frac{\sqrt{4-2ꙍ}+\sqrt{5-2ꙍ}}{\sqrt{\frac{1+ꙍ}{2}}+\sqrt{\frac{3+ꙍ}{2}}}\right)\right\}\right]}^{\frac{2}{3}}\leq {\left(\frac{1}{20}\left({8\left(4-2ꙍ\right)}^{\frac{5}{2}}-{\sqrt{2}\left(ꙍ+1\right)}^{\frac{5}{2}}\right)\right)}^{\frac{2}{3}}+{\left(\frac{1}{4}\left({16\left(ꙍ-2\right)}^{4}-{\frac{1}{16}\left(ꙍ+1\right)}^{4}\right)\right)}^{\frac{2}{3}}\text{,}$$

for each ꙍ∈[0,1].

Hence, Minkowski-like inequality (24) is satisfied. For (25), we have

$${\left[\int_{s-ᴫ\left(1-ꙍ\right)}^{t+\mu \left(1-ꙍ\right)}{\left(\Upsilon \left(ⱷ\right)\right)}^{p}dⱷ\right]}^{\frac{1}{p}}={\left(\frac{1}{6}\left({4\left(4-2ꙍ\right)}^{\frac{3}{2}}-{\sqrt{2}\left(ꙍ+1\right)}^{\frac{3}{2}}\right)\right)}^{2}\text{,}$$

$${\left[\int_{s-ᴫ\left(1-ꙍ\right)}^{t+\mu \left(1-ꙍ\right)}{\left(\varphi \left(ⱷ\right)\right)}^{p}dⱷ\right]}^{\frac{1}{p}}={\left(\frac{3}{8}\left({5ꙍ}^{2}-22ꙍ+21\right)\right)}^{2}\text{,}$$

$${\left(\int_{s-ᴫ\left(1-ꙍ\right)}^{t+\mu \left(1-ꙍ\right)}{\left(\Upsilon \left(ⱷ\right)+\varphi \left(ⱷ\right)\right)}^{p}dⱷ\right)}^{\frac{1}{p}}={\left[\frac{1}{4}\left\{\sqrt{\left(4-2ꙍ\right)\left(5-2ꙍ\right)}\left(9-4ꙍ-\frac{\sinh^{-1}\left(\sqrt{4-2ꙍ}\right)}{\sqrt{\left(4-2ꙍ\right)\left(5-2ꙍ\right)}}\right)-\sqrt{\left(\frac{1+ꙍ}{2}\right)\left(\frac{3+ꙍ}{2}\right)}\left(2+ꙍ-\frac{\sinh^{-1}\left(\sqrt{\frac{1+ꙍ}{2}}\right)}{\sqrt{\left(\frac{1+ꙍ}{2}\right)\left(\frac{3+ꙍ}{2}\right)}}\right)\right\}\right]}^{2}\text{.}$$

Then,

$${\left[\frac{1}{4}\left\{\sqrt{\left(4-2ꙍ\right)\left(5-2ꙍ\right)}\left(9-4ꙍ-\frac{\sinh^{-1}\left(\sqrt{4-2ꙍ}\right)}{\sqrt{\left(4-2ꙍ\right)\left(5-2ꙍ\right)}}\right)-\sqrt{\left(\frac{1+ꙍ}{2}\right)\left(\frac{3+ꙍ}{2}\right)}\left(2+ꙍ-\frac{\sinh^{-1}\left(\sqrt{\frac{1+ꙍ}{2}}\right)}{\sqrt{\left(\frac{1+ꙍ}{2}\right)\left(\frac{3+ꙍ}{2}\right)}}\right)\right\}\right]}^{2}$$

$$\geq {\left(\frac{1}{6}\left({4\left(4-2ꙍ\right)}^{\frac{3}{2}}-{\sqrt{2}\left(ꙍ+1\right)}^{\frac{3}{2}}\right)\right)}^{2}+{\left(\frac{3}{8}\left({5ꙍ}^{2}-22ꙍ+21\right)\right)}^{2}\text{,}$$

for each ꙍ∈[0,1].

Hence, Minkowski-like inequality (25) is satisfied.

$$\frac{\frac{1}{64}\left\{\sqrt{\left(4-2ꙍ\right)\left(5-2ꙍ\right)}\left(9-4ꙍ\right))\left(16\left(2-ꙍ\right)\left(5-2ꙍ\right)-3\right)-\sqrt{\frac{1+ꙍ}{2}\left(\frac{3+ꙍ}{2}\right)}\left(2+ꙍ\right)\left(4\left(1+ꙍ\right)\left(\frac{3+ꙍ}{2}\right)-3\right)+3\;\ln\left(\frac{\sqrt{4-2ꙍ}+\sqrt{5-2ꙍ}}{\sqrt{\frac{1+ꙍ}{2}}+\sqrt{\frac{3+ꙍ}{2}}}\right)\right\}}{\frac{1}{4}\left\{\sqrt{\left(4-2ꙍ\right)\left(5-2ꙍ\right)}\left(9-4ꙍ-\frac{\sinh^{-1}\left(\sqrt{4-2ꙍ}\right)}{\sqrt{\left(4-2ꙍ\right)\left(5-2ꙍ\right)}}\right)-\sqrt{\left(\frac{1+ꙍ}{2}\right)\left(\frac{3+ꙍ}{2}\right)}\left(2+ꙍ-\frac{\sinh^{-1}\left(\sqrt{\frac{1+ꙍ}{2}}\right)}{\sqrt{\left(\frac{1+ꙍ}{2}\right)\left(\frac{3+ꙍ}{2}\right)}}\right)\right\}}$$

$$\leq \frac{\frac{1}{20}\left({8\left(4-2ꙍ\right)}^{\frac{5}{2}}-{\sqrt{2}\left(ꙍ+1\right)}^{\frac{5}{2}}\right)}{\frac{1}{6}\left({4\left(4-2ꙍ\right)}^{\frac{3}{2}}-{\sqrt{2}\left(ꙍ+1\right)}^{\frac{3}{2}}\right)}+\frac{\frac{1}{4}\left({16\left(ꙍ-2\right)}^{4}-{\frac{1}{16}\left(ꙍ+1\right)}^{4}\right)}{\frac{3}{8}\left({5ꙍ}^{2}-22ꙍ+21\right)}\text{,}$$

for each ꙍ∈[0,1].

## 7 Applications of Hölder's-like inequality under convex-like real-valued mapping over fuzzy domain

Before starting the main outcomes of this section, firstly, we recall the identity that is recently introduced by Khan and Guirao [43] such that.

Lemma 1 Let $\Upsilon :\tilde{Ʊ}\to R$ be a real-valued mapping on $\tilde{Ʊ}\text{,}$ whose parametrized form is ${\left[\tilde{Ʊ}\right]}^{ꙍ}=\left[ʌ\left(ꙍ\right),ʋ\left(ꙍ\right)\right]$, for all ꙍ∈[0,1]. If Υ is differentiable on (ʌ(ꙍ),ʋ(ꙍ)) and $\Upsilon \in L_{P}\left[\tilde{Ʊ}\right]$, then the following inequality hold

$$\frac{\Upsilon \left(ʌ\left(ꙍ\right)\right)+\Upsilon \left(ʋ\left(ꙍ\right)\right)}{2}-\frac{1}{ʋ\left(ꙍ\right)-ʌ\left(ꙍ\right)}\int_{ʌ\left(ꙍ\right)}^{ʋ\left(ꙍ\right)}\Upsilon \left(ⱷ\right)dⱷ$$

(36) $$=\frac{ʋ\left(ꙍ\right)-ʌ\left(ꙍ\right)}{2}\int_{0}^{1}\left(1-2ʎ\right)\Upsilon^{\prime }\left(ʎʌ\left(ꙍ\right)+\left(1-ʎ\right)ʋ\left(ꙍ\right)\right)dʎ\text{.}$$

For convex-like real-valued mappings, some applications of identity (36) related to Hermite-Hadamard's integral inequality have been discussed in [43]. Here, we will present a few additional and intriguing examples by using Hölder's-like integral inequality.

Theorem 7 Let $\Upsilon :\tilde{Ʊ}\to R$ be a real-valued mapping on $\tilde{Ʊ}\text{,}$ whose parametrized form is ${\left[\tilde{Ʊ}\right]}^{ꙍ}=\left[ʌ\left(ꙍ\right),ʋ\left(ꙍ\right)\right]$, for all ꙍ∈[0,1]. Let p≥1 and Υ be differentiable on ${\tilde{Ʊ}}^{\circ }$ with ʋ(ꙍ)>ʌ(ꙍ). If ${\left|\Upsilon^{\prime }\right|}^{\frac{p}{p-1}}$ is convex-like mapping on $\tilde{Ʊ}$, then the following inequality hold

(37) $$\left|\frac{\Upsilon \left(ʌ\left(ꙍ\right)\right)+\Upsilon \left(ʋ\left(ꙍ\right)\right)}{2}-\frac{1}{ʋ\left(ꙍ\right)-ʌ\left(ꙍ\right)}\int_{ʌ\left(ꙍ\right)}^{ʋ\left(ꙍ\right)}\Upsilon \left(ⱷ\right)dⱷ\right|\leq \frac{ʋ\left(ꙍ\right)-ʌ\left(ꙍ\right)}{2{\left(p+1\right)}^{\frac{1}{p}}}{\left[\frac{{\left|\Upsilon^{\prime }\left(ʌ\left(ꙍ\right)\right)\right|}^{\frac{p}{p-1}}+{\left|\Upsilon^{\prime }\left(ʋ\left(ꙍ\right)\right)\right|}^{\frac{p}{p-1}}}{2}\right]}^{\frac{p-1}{p}}\text{.}$$

**Proof**. With the help of Lemma 1 and Hölder's-like integral inequality, since ${\left[\tilde{Ʊ}\right]}^{0}=\left[ʌ\left(0\right),ʋ\left(0\right)\right]$ (and hence on every ${\left[\tilde{Ʊ}\right]}^{ꙍ}$, for all ꙍ∈[0,1]) we find

$$\left|\frac{\Upsilon \left(ʌ\left(0\right)\right)+\Upsilon \left(ʋ\left(0\right)\right)}{2}-\frac{1}{ʋ\left(0\right)-ʌ\left(0\right)}\int_{ʌ\left(0\right)}^{ʋ\left(0\right)}\;\Upsilon \left(ⱷ\right)dⱷ\right|\leq \frac{ʋ\left(0\right)-ʌ\left(0\right)}{2}\int_{0}^{1}\;\left|1-2ʎ\right|\left|\Upsilon^{\prime }\left(ʎʌ\left(0\right)+\left(1-ʎ\right)ʋ\left(0\right)\right)\right|dʎ$$

(38) $$\leq \frac{ʋ\left(0\right)-ʌ\left(0\right)}{2}{\left(\int_{0}^{1}\;|1-2ʎ{|}^{p}dʎ\right)}^{1/p}{\left(\int_{0}^{1}\;{\left|\Upsilon^{\prime }\left(ʎʌ\left(0\right)+\left(1-ʎ\right)ʋ\left(0\right)\right)\right|}^{q}dʎ\right)}^{1/q}\text{,}$$

where 1/p+1/q=1.

Since ${\left|\Upsilon^{\prime }\right|}^{q}$ convex-like mapping on $\tilde{Ʊ}$, we have

$$\int_{0}^{1}\;{\left|\Upsilon^{\prime }\left(ʎʌ\left(0\right)+\left(1-ʎ\right)ʋ\left(0\right)\right)\right|}^{q}dʎ\leq \int_{0}^{1}\;\left[ʎ{\left|\Upsilon^{\prime }\left(ʌ\left(0\right)\right)\right|}^{q}+\left(1-ʎ\right){\left|\Upsilon^{\prime }\left(ʋ\left(0\right)\right)\right|}^{q}\right]dʎ$$

(39) $$=\frac{{\left|\Upsilon^{\prime }\left(ʌ\left(0\right)\right)\right|}^{q}+{\left|\Upsilon^{\prime }\left(ʋ\left(0\right)\right)\right|}^{q}}{2}\text{.}$$

Further, since

(40) $$\int_{0}^{1}\;|1-2ʎ{|}^{p}dʎ=\int_{0}^{1/2}\;{\left(1-2ʎ\right)}^{p}dʎ+\int_{1/2}^{1}\;{\left(2ʎ-1\right)}^{p}dʎ=2\int_{0}^{1/2}\;{\left(1-2ʎ\right)}^{p}dʎ=\frac{1}{p+1}\text{.}$$

From (38) and (39), the inequality (37), we have

$$\left|\frac{\Upsilon \left(ʌ\left(0\right)\right)+\Upsilon \left(ʋ\left(0\right)\right)}{2}-\frac{1}{ʋ\left(0\right)-ʌ\left(0\right)}\int_{ʌ\left(0\right)}^{ʋ\left(0\right)}\;\Upsilon \left(ⱷ\right)dⱷ\right|\leq \frac{ʋ\left(0\right)-ʌ\left(0\right)}{2}\int_{0}^{1}\;\left|1-2ʎ\right|\left|\Upsilon^{\prime }\left(ʎʌ\left(0\right)+\left(1-ʎ\right)ʋ\left(0\right)\right)\right|dʎ$$

$$\leq \frac{ʋ\left(0\right)-ʌ\left(0\right)}{2{\left(p+1\right)}^{\frac{1}{p}}}{\left[\frac{{\left|\Upsilon^{\prime }\left(ʌ\left(0\right)\right)\right|}^{\frac{p}{p-1}}+{\left|\Upsilon^{\prime }\left(ʋ\left(0\right)\right)\right|}^{\frac{p}{p-1}}}{2}\right]}^{\frac{p-1}{p}}\text{.}$$

For every ${\left[\tilde{Ʊ}\right]}^{ꙍ}$, for all ꙍ∈[0,1], we have

$$\left|\frac{\Upsilon \left(ʌ\left(ꙍ\right)\right)+\Upsilon \left(ʋ\left(ꙍ\right)\right)}{2}-\frac{1}{ʋ\left(ꙍ\right)-ʌ\left(ꙍ\right)}\int_{ʌ\left(ꙍ\right)}^{ʋ\left(ꙍ\right)}\Upsilon \left(ⱷ\right)dⱷ\right|\leq \frac{ʋ\left(ꙍ\right)-ʌ\left(ꙍ\right)}{2{\left(p+1\right)}^{\frac{1}{p}}}{\left[\frac{{\left|\Upsilon^{\prime }\left(ʌ\left(ꙍ\right)\right)\right|}^{\frac{p}{p-1}}+{\left|\Upsilon^{\prime }\left(ʋ\left(ꙍ\right)\right)\right|}^{\frac{p}{p-1}}}{2}\right]}^{\frac{p-1}{p}}\text{,}$$

hence, the required result.

Theorem 8 Let $\Upsilon :\tilde{Ʊ}\to R$ be a real-valued mapping on $\tilde{Ʊ}\text{,}$ whose parametrized form is ${\left[\tilde{Ʊ}\right]}^{ꙍ}=\left[ʌ\left(ꙍ\right),ʋ\left(ꙍ\right)\right]$, for all ꙍ∈[0,1]. Let q≥1 and Υ be differentiable on ${\tilde{Ʊ}}^{\circ }$. If ${\left|\Upsilon^{\prime }\right|}^{q}$ is convex-like mapping on $\tilde{Ʊ}$, then the following inequality hold

(41) $$\left|\frac{\Upsilon \left(ʌ\left(ꙍ\right)\right)+\Upsilon \left(ʋ\left(0\right)\right)}{2}-\frac{1}{ʋ\left(ꙍ\right)-ʌ\left(ꙍ\right)}\int_{ʌ\left(ꙍ\right)}^{ʋ\left(ꙍ\right)}\;\Upsilon \left(ⱷ\right)dⱷ\right|\leq \frac{ʋ\left(ꙍ\right)-ʌ\left(ꙍ\right)}{4}{\left[\frac{{\left|\Upsilon^{\prime }\left(ʌ\left(ꙍ\right)\right)\right|}^{q}+{\left|\Upsilon^{\prime }\left(ʋ\left(ꙍ\right)\right)\right|}^{q}}{2}\right]}^{1/q}\text{.}$$

Proof. From Lemma 1

(42) $$\left|\frac{\Upsilon \left(ʌ\left(0\right)\right)+\Upsilon \left(ʋ\left(0\right)\right)}{2}-\frac{1}{ʋ\left(0\right)-ʌ\left(0\right)}\int_{ʌ\left(0\right)}^{ʋ\left(0\right)}\;\Upsilon \left(ⱷ\right)dⱷ\right|\leq \frac{ʋ\left(0\right)-ʌ\left(0\right)}{2}\int_{0}^{1}\;\left|1-2ʎ\right|\left|\Upsilon^{\prime }\left(ʎʌ\left(0\right)+\left(1-ʎ\right)ʋ\left(0\right)\right)\right|dʎ\text{,}$$

hence on every ${\left[\tilde{Ʊ}\right]}^{ꙍ}$, for all ꙍ∈[0,1] and by the power-mean inequality

$$\begin{array}{rr} & \;\int_{0}^{1}\;\left|1-2ʎ\right|\left|\Upsilon^{\prime }\left(ʎʌ\left(0\right)+\left(1-ʎ\right)ʋ\left(0\right)\right)\right|dʎ\\ & \leq {\left(\int_{0}^{1}\;\left|1-2ʎ\right|dʎ\right)}^{1-1/q}{\left(\int_{0}^{1}\;\left|1-2ʎ\right|{\left|\Upsilon^{\prime }\left(ʎʌ\left(0\right)+\left(1-ʎ\right)ʋ\left(0\right)\right)\right|}^{q}dʎ\right)}^{1/q} \end{array}$$

Because ${\left|\Upsilon^{\prime }\right|}^{q}$ is convex-like mapping, we have

$$\begin{array}{rr} \int_{0}^{1}\;\left|1-2ʎ\right|{\left|\Upsilon^{\prime }\left(ʎʌ\left(0\right)+\left(1-ʎ\right)ʋ\left(0\right)\right)\right|}^{q}dʎ & \leq \int_{0}^{1}\;\left|1-2ʎ\right|\left[ʎ{\left|\Upsilon^{\prime }\left(ʌ\left(0\right)\right)\right|}^{q}+\left(1-ʎ\right){\left|\Upsilon^{\prime }\left(ʋ\left(0\right)\right)\right|}^{q}\right]dʎ\\ & =\frac{{\left|\Upsilon^{\prime }\left(ʌ\left(0\right)\right)\right|}^{q}+{\left|\Upsilon^{\prime }\left(ʋ\left(0\right)\right)\right|}^{q}}{4} \end{array}$$

Since $\int_{0}^{1}\;\left|1-2ʎ\right|dʎ=1/2$, we have from (42) and the displayed inequality following it that

$$\left|\frac{\Upsilon \left(ʌ\left(0\right)\right)+\Upsilon \left(ʋ\left(0\right)\right)}{2}-\frac{1}{ʋ\left(0\right)-ʌ\left(0\right)}\int_{ʌ\left(0\right)}^{ʋ\left(0\right)}\;\Upsilon \left(ⱷ\right)dⱷ\right|\leq \frac{ʋ\left(0\right)-ʌ\left(0\right)}{2}{\left(\frac{1}{2}\right)}^{1-1/q}{\left(\frac{{\left|\Upsilon^{\prime }\left(ʌ\left(0\right)\right)\right|}^{q}+{\left|\Upsilon^{\prime }\left(ʋ\left(0\right)\right)\right|}^{q}}{4}\right)}^{1/q}$$

hence, the desired result.

We now move on to a comparable finding concerning the Hermite-Hadamard inequality such that.

Theorem 9 Suppose the assumptions of Theorem 8 are satisfied. Then

(43) $$\left|\Upsilon \left(\frac{ʌ\left(ꙍ\right)+ʋ\left(ꙍ\right)}{2}\right)-\frac{1}{ʋ\left(ꙍ\right)-ʌ\left(ꙍ\right)}\int_{ʌ\left(ꙍ\right)}^{ʋ\left(ꙍ\right)}\;\Upsilon \left(ⱷ\right)dⱷ\right|\leq \frac{ʋ\left(ꙍ\right)-ʌ\left(ꙍ\right)}{4}{\left[\frac{{\left|\Upsilon^{\prime }\left(ʌ\left(ꙍ\right)\right)\right|}^{q}+{\left|\Upsilon^{\prime }\left(ʋ\left(ꙍ\right)\right)\right|}^{q}}{2}\right]}^{1/q}\text{.}$$

**Proof**. Our starting point is the identity

(44) $$\Upsilon \left(\frac{ʌ\left(0\right)+ʋ\left(0\right)}{2}\right)-\frac{1}{ʋ\left(0\right)-ʌ\left(0\right)}\int_{ʌ\left(0\right)}^{ʋ\left(0\right)}\;\Upsilon \left(ⱷ\right)dⱷ=\frac{1}{ʋ\left(0\right)-ʌ\left(0\right)}\left[\int_{ʌ\left(0\right)}^{\frac{ʌ\left(0\right)+ʋ\left(0\right)}{2}}\;\left(ⱷ-ʌ\left(0\right)\right)\Upsilon^{\prime }\left(ⱷ\right)dⱷ+\int_{\frac{ʌ\left(0\right)+ʋ\left(0\right)}{2}}^{ʋ\left(0\right)}\;\left(ⱷ-ʋ\left(0\right)\right)\Upsilon^{\prime }\left(ⱷ\right)dⱷ\right]\text{,}$$

and hence on every ${\left[\tilde{Ʊ}\right]}^{ꙍ}$, for all ꙍ∈[0,1]. The desired result is obtained by an argument similar to that of Theorem 8, but with (44) in place of Lemma 1.

Applications to special means

For arbitrary fuzzy numbers, relatively few results are known. We can utilize some of the aforementioned means in the following ways:

$$\begin{array}{rrrr} A\left(ɤ,\theta \right) & =\frac{ɤ+\theta }{2}\text{,} & & ɤ,\theta \in {\left[\tilde{Ʊ}\right]}^{0}\text{,}\\ \bar{L}\left(ɤ,\theta \right) & =\frac{\theta -ɤ}{\ln\;|\theta \left|-\ln\;\right|ɤ|}\text{,} & & ɤ,\theta \in {\left[\tilde{Ʊ}\right]}^{0}\setminus \left\{0\right\}\text{,}\\ L_{n}\left(ɤ,\theta \right) & ={\left[\frac{\theta^{n+1}-{ɤ}^{n+1}}{\left(n+1\right)\left(\theta -ɤ\right)}\right]}^{1/n}\text{,} & & n\in N,n\geq 1,ɤ,\theta \in {\left[\tilde{Ʊ}\right]}^{0},ɤ<\theta \end{array}$$

and hence on every ${\left[\tilde{Ʊ}\right]}^{ꙍ}$, for all ꙍ∈[0,1].

Proposition 1 Let ${\left[\tilde{Ʊ}\right]}^{ꙍ}$ be fuzzy number, and n∈N,n≥2. Then, for all p>1, the following inequality holds:

$$\left|A\left({\left(ʌ\left(ꙍ\right)\right)}^{n},{\left(ʋ\left(ꙍ\right)\right)}^{n}\right)-L_{n}\left(ʌ\left(ꙍ\right),ʋ\left(ꙍ\right)\right)\right|$$

(45) $$\leq \frac{n\left(ʋ\left(ꙍ\right)-ʌ\left(ꙍ\right)\right)}{2{\left(p+1\right)}^{1/p}}{\left[A\left(|ʌ\left(ꙍ\right){|}^{\left(n-1\right)p/\left(p-1\right)},|ʋ\left(ꙍ\right){|}^{\left(n-1\right)p/\left(p-1\right)}\right)\right]}^{\left(p-1\right)/p}\text{.}$$

**Proof**. The proof is immediate from Theorem 7 applied for $\Upsilon \left(ⱷ\right)={ⱷ}^{n},ⱷ\in {\left[\tilde{Ʊ}\right]}^{ꙍ}$.

Proposition 2 Let 0∉ ${\left[\tilde{Ʊ}\right]}^{0}=\left[ʌ\left(0\right),ʋ\left(0\right)\right]$ (and hence on every ${\left[\tilde{Ʊ}\right]}^{ꙍ}$, for all ꙍ∈[0,1]). Then, the following inequality holds:

(46) $$\left|A\left({\left(ʌ\left(ꙍ\right)\right)}^{-1},{\left(ʋ\left(ꙍ\right)\right)}^{-1}\right)-{\bar{L}}^{-1}\left(ʌ\left(ꙍ\right),ʋ\left(ꙍ\right)\right)\right|\leq \frac{\left(ʋ\left(ꙍ\right)-ʌ\left(ꙍ\right)\right)}{4}A\left(|ʌ\left(ꙍ\right){|}^{-2},|ʋ\left(ꙍ\right){|}^{-2}\right)\text{.}$$

**Proof**. The proof is obvious from Theorem 7 applied for $\Upsilon \left(ⱷ\right)=1/ⱷ,ⱷ\in {\left[\tilde{Ʊ}\right]}^{ꙍ}$.

Proposition 3 Let $0\notin {\left[\tilde{Ʊ}\right]}^{0}=\left[ʌ\left(0\right),ʋ\left(0\right)\right]$ (and hence on every ${\left[\tilde{Ʊ}\right]}^{ꙍ}$, for all ꙍ∈[0,1]). Then, for p>1, the following inequality holds:

$$\left|A\left({\left(ʌ\left(ꙍ\right)\right)}^{-1},{\left(ʋ\left(ꙍ\right)\right)}^{-1}\right)-{\bar{L}}^{-1}\left(ʌ\left(ꙍ\right),ʋ\left(ꙍ\right)\right)\right|$$

(47) $$\leq \frac{\left(ʋ\left(ꙍ\right)-ʌ\left(ꙍ\right)\right)}{2{\left(p+1\right)}^{1/p}}{\left[A\left(|ʌ\left(ꙍ\right){|}^{-2p/\left(p-1\right)},|ʋ\left(ꙍ\right){|}^{-2p/\left(p-1\right)}\right)\right]}^{\left(p-1\right)/p}\text{.}$$

**Proof**. The proof is obvious from Theorem 7 applied for $\Upsilon \left(ⱷ\right)=1/ⱷ,ⱷ\in {\left[\tilde{Ʊ}\right]}^{ꙍ}$.

The next results are extensions of Proposition 1, Proposition 2, Proposition 3 such that.

Proposition 4 Let $0\notin {\left[\tilde{Ʊ}\right]}^{0}=\left[ʌ\left(0\right),ʋ\left(0\right)\right]$ (and hence on every ${\left[\tilde{Ʊ}\right]}^{ꙍ}$, for all ꙍ∈[0,1]), and n∈Z,|n|≥2. Then for all q≥1,

(48) $$\left|A\left({\left(ʌ\left(ꙍ\right)\right)}^{n},{\left(ʋ\left(ꙍ\right)\right)}^{n}\right)-L_{n}{\left(ʌ\left(ꙍ\right),ʋ\left(ꙍ\right)\right)}^{n}\right|\leq \frac{|n|\left(ʋ\left(ꙍ\right)-ʌ\left(ꙍ\right)\right)}{4}{\left[A\left(|ʌ\left(ꙍ\right){|}^{\left(n-1\right)q},|ʋ\left(ꙍ\right){|}^{\left(n-1\right)q}\right)\right]}^{1/q}\text{.}$$

and

(49) $$\left|A{\left(ʌ\left(ꙍ\right),ʋ\left(ꙍ\right)\right)}^{n}-L_{n}{\left(ʌ\left(ꙍ\right),ʋ\left(ꙍ\right)\right)}^{n}\right|\leq \frac{|n|\left(ʋ\left(ꙍ\right)-ʌ\left(ꙍ\right)\right)}{4}{\left[A\left(|ʌ\left(ꙍ\right){|}^{\left(n-1\right)q},|ʋ\left(ꙍ\right){|}^{\left(n-1\right)q}\right)\right]}^{1/q}\text{.}$$

**Proof**. The proof is immediate from Theorem 8 and Theorem 9 with $\Upsilon \left(ⱷ\right)={ⱷ}^{n},ⱷ\in R,n\in Z$, n≥2.

Proposition 5 Suppose $0\notin {\left[\tilde{Ʊ}\right]}^{0}=\left[ʌ\left(0\right),ʋ\left(0\right)\right]$ (and hence on every ${\left[\tilde{Ʊ}\right]}^{ꙍ}$, for all ꙍ∈[0,1]). Then for q≥1,

(50) $$\left|A\left({\left(ʌ\left(ꙍ\right)\right)}^{-1},{\left(ʋ\left(ꙍ\right)\right)}^{-1}\right)-L^{-1}\left(ʌ\left(ꙍ\right),ʋ\left(ꙍ\right)\right)\right|\leq \frac{ʋ\left(ꙍ\right)-ʌ\left(ꙍ\right)}{4}{\left[A\left(|ʌ\left(ꙍ\right){|}^{-2q},|ʋ\left(ꙍ\right){|}^{-2q}\right)\right]}^{1/q}\text{,}$$

and

(51) $$\left|A{\left(ʌ\left(ꙍ\right),ʋ\left(ꙍ\right)\right)}^{-1}-L^{-1}\left(ʌ\left(ꙍ\right),ʋ\left(ꙍ\right)\right)\right|\leq \frac{\left(ʋ\left(ꙍ\right)-ʌ\left(ꙍ\right)\right)}{4}{\left[A\left(|ʌ\left(ꙍ\right){|}^{-2q},|ʋ\left(ꙍ\right){|}^{-2q}\right)\right]}^{1/q}\text{.}$$

**Proof**. The result follows from Theorem 8 and Theorem 9 with Υ(ⱷ)=1/ⱷ.

## 8 Conclusion

In this paper, using a straightforward proof method over newly defined $L_{P}$ space, we established several new refinements for integral forms of classical Hölder's and newly defined Hölder's-like inequality. Numerous existing inequalities linked with Hölder's-like inequality over fuzzy domain can be improved through the newly obtained ones, as illustrated through applications like Hölder's power-mean-like integral inequality, Cauchy-Schwarz-like inequality, Minkowski's-like inequality, and Beckenbach's-like inequality over fuzzy domain. Additionally, by using special means, some new versions of integral inequalities have obtained where differentiable mappings are real-valued convex-like mappings. Our discoveries represent significant progressions in the field of mathematics.

## CRediT authorship contribution statement

**Xiangting Shi:** Validation, Resources, Funding acquisition. **Ahmad Aziz Al Ahmadi:** Resources, Investigation, Funding acquisition, Formal analysis, Data curation. **Muhammad Bilal Khan:** Writing – review & editing, Writing – original draft, Visualization, Validation, Supervision, Resources, Project administration, Investigation, Conceptualization. **Loredana Ciurdariu:** Validation, Resources, Methodology, Formal analysis, Data curation. **Khalil Hadi Hakami:** Visualization, Validation, Software, Resources, Funding acquisition, Formal analysis, Data curation.

## Data availability statement

Not applicable.

## Funding

The authors extend their appreciation to 10.13039/501100006261Taif University, Saudi Arabia, for supporting this work through project number (TU-DSPP-2024-56).

## Declaration of competing interest

The authors declare the following financial interests/personal relationships which may be considered as potential competing interests: Loredana Ciurdariu reports article publishing charges was provided by Politehnica University Timisoara Department of Mathematics. If there are other authors, they declare that they have no known competing financial interests or personal relationships that could have appeared to influence the work reported in this paper.
